# A30 MICROBIOTA AND NOD2 REGULATE SMALL INTESTINAL RESTITUTION THROUGH FETAL-LIKE INTESTINAL STEM CELLS

**DOI:** 10.1093/jcag/gwad061.030

**Published:** 2024-02-14

**Authors:** D Tsang, C Maisonneuve, A Ayyaz, E Foerster, M Nissan, L Baerg, D Trcka, C Streutker, J L Wrana, S E Girardin, D Philpott

**Affiliations:** . Department of Immunology, University of Toronto, Toronto, ON, Canada; . Department of Immunology, University of Toronto, Toronto, ON, Canada; . University of Calgary, Calgary, AB, Canada; . Department of Immunology, University of Toronto, Toronto, ON, Canada; . Department of Laboratory Medicine and Pathobiology, University of Toronto, Toronto, ON, Canada; . Department of Chemical Engineering and Applied Chemistry, University of Toronto, Toronto, ON, Canada; . Lunenfeld-Tanenbaum Research Institute, Toronto, ON, Canada; . Unity Health Toronto, Toronto, ON, Canada; . Lunenfeld-Tanenbaum Research Institute, Toronto, ON, Canada; . Department of Laboratory Medicine and Pathobiology, University of Toronto, Toronto, ON, Canada; . Department of Immunology, University of Toronto, Toronto, ON, Canada

## Abstract

**Background:**

Inflammatory bowel disease is characterized by chronic inflammation of the gastrointestinal tract, resulting in recurrent injury to the intestinal epithelium. Restitution of the small intestinal epithelium is a coordinated response that involves the dedifferentiation of epithelial cell lineages, proliferation of Lgr5+ intestinal stem cells, and fetal-like stem cell reversion. While gut microbiota are critical mediators of intestinal inflammation, their impact on epithelial restitution remains unclear.

**Aims:**

We aim to identify the how microbes regulate small intestinal epithelial restitution following damage. Our hypothesis is that gut microbiota accelerate restitution through pattern recognition receptor-driven signals following fetal-like stem cell reversion.

**Methods:**

Irradiation (IR, 12Gγ) was used to induce a synchronized small intestinal epithelial restitution response in mice. Intestinal restitution kinetics were assessed transcriptionally (scRNA-Seq, qPCR) and histologically. Small intestinal organoids were used to assess epithelial restitution kinetics *in vitro*.

**Results:**

ScRNA-Seq of small intestinal epithelial cells following IR from germ-free mice (GF), and specific pathogen-free mice (SPF) mice revealed that microbiota induced greater expression of fetal-like stem cell reversion markers, *Ly6a* and *Clu,* and greater expression of the proliferation marker, *Pcna*. These results were supported histologically as irradiated SPF mice observed an increase in fetal-like stem cells marked by *Ly6a* and *Clu* and an increase BrdU+ proliferating cells. ScRNA-seq and *in situ* hybridization highlighted that fetal-like intestinal stem cells upregulate expression of *Nod2*, a bacterial pattern recognition receptor. Using intestinal organoids to assess the function of Nod2, we observed that muramyl dipeptide driven Nod2-signlaing potentiates an interferon gene signature following IFNγ and TNFα co-stimulation. Further supporting the role for Nod2 in intestinal restitution, intestinal epithelium specific Nod2KO mice decreased BrdU+ proliferating cells post-IR compared to littermate controls.

**Conclusions:**

Microbiota promote small intestinal restitution following IR through Nod2-signaling in fetal-like intestinal stem cells.

**Funding Agencies:**

CIHR

